# The Role of Peroxisome Proliferator-Activated Receptors (PPARs) in Pan-Cancer

**DOI:** 10.1155/2020/6527564

**Published:** 2020-09-22

**Authors:** Runzhi Huang, Jiaqi Zhang, Mingxiao Li, Penghui Yan, Huabin Yin, Suna Zhai, Xiaolong Zhu, Peng Hu, Jiaxin Zhang, Ling Huang, Man Li, Zehui Sun, Tong Meng, Daoke Yang, Zongqiang Huang

**Affiliations:** ^1^Department of Orthopedics, The First Affiliated Hospital of Zhengzhou University, 1 East Jianshe Road, Zhengzhou, China; ^2^Division of Spine, Department of Orthopedics, Tongji Hospital Affiliated to Tongji University School of Medicine, 389 Xincun Road, Shanghai, China; ^3^Tongji University School of Medicine, 1239 Siping Road, Shanghai 200092, China; ^4^Department of Orthopedics, Shanghai General Hospital, School of Medicine, Shanghai Jiaotong University, 100 Haining Road, Shanghai, China; ^5^Department of Radiotherpy, The First Affiliated Hospital of Zhengzhou University, Zhengzhou 450052, China; ^6^Tongji University Cancer Center, Shanghai Tenth People's Hospital of Tongji University, School of Medicine, Tongji University, Shanghai, China

## Abstract

Peroxisome proliferator-activated receptors (PPARs) are members of nuclear transcription factors. The functions of the PPAR family (PPARA, PPARD, and PPARG) and their coactivators (PPARGC1A and PPARGC1B) in maintenance of lipid and glucose homeostasis have been unveiled. However, the roles of PPARs in cancer development remain elusive. In this work, we made use of 11,057 samples across 33 TCGA tumor types to analyze the relationship between PPAR transcriptional expression and tumorigenesis as well as drug sensitivity. We performed multidimensional analyses on PPARA, PPARG, PPARD, PPARGC1A, and PPARGC1B, including differential expression analysis in pan-cancer, immune subtype analysis, clinical analysis, tumor purity analysis, stemness correlation analysis, and drug responses. PPARs and their coactivators expressed differently in different types of cancers, in different immune subtypes. This analysis reveals various expression patterns of the PPAR family at a level of pan-cancer and provides new clues for the therapeutic strategies of cancer.

## 1. Introduction

Peroxisome proliferator-activated receptors (PPARs), members of nuclear receptor subfamily, are a series of ligand-activated transcription factors (TFs) that regulate the expression of target genes, which involve in various biological processes, including cellular differentiation, cell proliferation, lipid metabolism, and tumorigenesis [1]. PPARs can be activated by various ligands, such as fatty acids (FAs), eicosanoids, and some targeted drugs [2]. Upon binding to the ligand, PPARs form a heterodimer with retinoid X receptor (RXR), and this PPAR/RXR complex is required for its subsequent binding to specific DNA regions in PPAR response elements (PPREs), the gene promotor region [3]. PPARs then trigger transcription of target genes after recruitment of coactivators and release of corepressors [4]. PPARGC1A and PPARGC1B were peroxisome proliferator-activated receptor gamma coactivators 1 alpha and beta, respectively, playing important roles in the PPAR signaling network [5]. There are mainly three isotypes of PPARs with distinct tissue distribution, metabolic patterns, and ligand specificity: PPAR*α*, PPAR*γ*, and PPAR*δ* [6]. Although the roles of the three isotypes played in carcinogenesis and chemoprevention have not been clearly characterized [7], some agonists of them have been used in clinical trials for years. There is no conclusions but controversial results regarding the antitumor functions of PPAR*α* and PPAR*γ* [8]. The characteristics of PPARs differ from each other, and different isotypes may have different impacts in different types of cancer. To date, there is no bioinformatics study systematically investigating the transcriptional levels of each PPAR in pan-cancer. Thus, it is of importance to study the PPARs' expression patterns in pan-cancer and exploit the potential of PPAR-targeted drugs when it comes to the treatment of differentially PPAR-expressed tumors.

In this study, we analyzed the expression signatures of PPARA, PPARD, PPARG, PPARGC1AA, and PPARGC1B in pan-cancer. Utilizing multidimensional correlation analysis, we found the associations between transcriptional levels of PPARs and stemness, tumor purity, and drug sensitivity across TCGA cancers.

## 2. Materials and Methods

### 2.1. Data Downloading and Preprocessing

On June 23, 2020, the gene expression profiles, phenotype information, and survival data of PARRA, PPARD, PPARG, PPARGC1A, and PPARGC1B in 33 types of TCGA tumor samples and adjacent tissues (a total of 11,057 samples) were downloaded from GDC TCGA sets in the UCSC Xena database (http://xena.ucsc.edu/) in formats of Fragments Per Kilobase per Million (FPKM) and HTSeq-Counts. Meanwhile, demographics, tumor information, and follow-up data of all patients were also extracted from the database.

The 33 types of TCGA tumors and abbreviations were as follows: adrenocortical carcinoma (ACC), Bladder Urothelial Carcinoma (BLCA), breast invasive carcinoma (BRCA), cervical squamous cell carcinoma and endocervical adenocarcinoma (CESC), Cholangiocarcinoma (CHOL), colon adenocarcinoma (COAD), Lymphoid Neoplasm Diffuse Large B-cell Lymphoma (DLBC), esophageal carcinoma (ESCA), glioblastoma multiforme (GBM), head and neck squamous cell carcinoma (HNSC), Kidney Chromophobe (KICH), kidney renal clear cell carcinoma (KIRC), kidney renal papillary cell carcinoma (KIRP), Acute Myeloid Leukemia (LAML), Brain Lower Grade Glioma (LGG), liver hepatocellular carcinoma (LIHC), lung adenocarcinoma (LUAD), lung squamous cell carcinoma (LUSC), Mesothelioma (MESO), ovarian serous cystadenocarcinoma (OV), pancreatic adenocarcinoma (PAAD), Pheochromocytoma and Paraganglioma (PCPG), prostate adenocarcinoma (PRAD), rectum adenocarcinoma (READ), Sarcoma (SARC), Skin Cutaneous Melanoma (SKCM), stomach adenocarcinoma (STAD), Testicular Germ Cell Tumors (TGCT), thyroid carcinoma (THCA), Thymoma (THYM), Uterine Corpus Endometrial Carcinoma (UCEC), Uterine Carcinosarcoma (UCS), and Uveal Melanoma (UVM) from GDC TCGA documents in the UCSC Xena database.

### 2.2. Differential Expression Analysis and Coexpression Analysis of PPARs between Tumor and Normal Samples

For each and across all TCGA tumor types, we used the “ggpubr” R package to perform differential expression analysis (Wilcox test) between tumor and normal tissues. Only tumor types with more than 3 normal samples were included. The differences in expression of the 5 PPAR family genes in pan-cancer were presented in a form of log2 Fold Change (log2 FC) in a heatmap.

Using corrplot R package, coexpression analysis between PPARA, PPARD, PPARG, PPARGC1B, and PPARGC2B was also done at a transcriptional level, to explore the potential expression pattern between every two PPAR genes. Moreover, a protein-protein interaction network among those genes was constructed by using the STRING database (https://string-db.org/) [9].

### 2.3. Clinical Correlation Analysis

To analyze the differences in overall survival outcomes between patients expressing high and low levels of PPARs, Kaplan-Meier plots for PPAR genes in pan-cancer were generated by using the R package. Phenotype and survival data for 33 TCGA cancer types were downloaded on June 23, 2020, from GDC TCGA sets in the UCSC Xena database (http://xena.ucsc.edu/). Patients were divided into high- and low- expression groups according to the median expression level of PPARA, PPARD, PPARGC1A, and PPARGC1B, respectively.

In addition, Cox proportional hazard regression was applied to access the hazard ratios of PPARA, PPARD, PPARG, PPARGC1A, and PPARGC1B in each TCGA tumor type. Moreover, differential analysis was also used to detect the differences in the level of PPAR expression signatures in different stages of STAD as an example. The threshold for significance was set as two-paired *p* < 0.05.

### 2.4. Immune Subtype Analysis

Roles of immune tumor microenvironment (TME) were of therapeutic and prognostic significance in antitumor therapies. Six immune subtypes across TCGA tumor types had been identified by investigators based on five representative immune signatures, which offered a resource for analyzing the TME of some specific tumor. For TCGA tumors, the distribution of immune subtypes varies from each other and each immune subtype presents different biological and clinical features, which determine antitumor therapied to some extent [10]. To access the mRNA expression levels of PPARA, PPARD, PPARG, PPARGC1A, and PPARGC2B in the six different immune subtypes across TGCA tumor types, we performed differential expression analysis with the Kruskal test. Tumors were characterized by immunogenomic features identified by Thorsson et al., including wound healing (C1), IFN-*γ* dominant (C2), inflammatory (C3), lymphocyte depleted (C4), immunologically quiet (C5), and TGF-*β* dominant (C6) [10].

### 2.5. Stemness Indices and TME in Pan-Cancer

More than tumor cells, solid tumor tissues consist of other normal cells, such as stromal cells, immune cells, and vascular cells, which made up TME together. We intended to analyze the correlation between PPAR expression and the fraction of stromal and immune cells in TCGA tumor samples. Methods to access the proportion of these two TME components had been proposed, one of which was ESTIMATE (Estimation of STormal and Immune cells in MAlignant Tumors using Expression data) [11]. The ESTIMATE score was calculated based on gene expression signatures and could reflect tumor purity with favorable prediction accuracy. Thus, Spearman correlation analysis was performed between the expression level of 5 PPAR genes and stromal score by using the estimate package and limma package.

To further analyze the associations between PPARs and stemness features of pan-cancer, we calculated the stemness indices of TCGA tumor samples by using a one-class logistic regression (OCLR) algorithm and performed Spearman correlation analysis based on gene expression and stemness scores [12]. Stemness indices describe the features of self-renewal and dedifferentiation within tumor cells, which might promote distant metastasis and tumorigenesis. Here, two types of stemness indices were obtained, including the DNA methylation-based stemness index (DNAss) and mRNA expression-based stemness index (RNAss).

For breast invasive carcinoma and liver hepatocellular carcinoma, specifically, we accessed RNAss, DNAss, stromal score, immune score, and ESTIMATE score (the algebraic sum of the stromal score and the immune score) to analyze the correlation relationship with PPAR transcriptional expression.

### 2.6. Drug Sensitivity Analysis in Pan-Cancer

The data including the RNA-seq profiles of PPAR genes and the drug activity were downloaded from the CellMiner database (https://discover.nci.nih.gov/cellminer/). Impute package from Bioconductor (http://www.bioconductor.org/packages/release/bioc/html/impute.html) was used to preprocess the raw data. CellMiner is a web-based tool with genomic and pharmacologic information for investigators to make use of transcript and drug response data in the NCI-60 cell line sets, which was compiled by the U.S. National Cancer Institute [13]. Transcript expression levels of 22,379 genes, 360 microRNAs, and drug responses of 20,503 compounds are available in the CellMiner website [14]. To explore the correlation between the transcriptional expression of PPAR genes and compound sensitivity, we followed the methods of Dong et al. [15], and Pearson correlation analysis was performed between the two controlled by *p* value < 0.05.

## 3. Results

### 3.1. Differential Expression Analysis and Coexpression Analysis of PPARs between Tumor and Normal Samples

**Figure 1:**
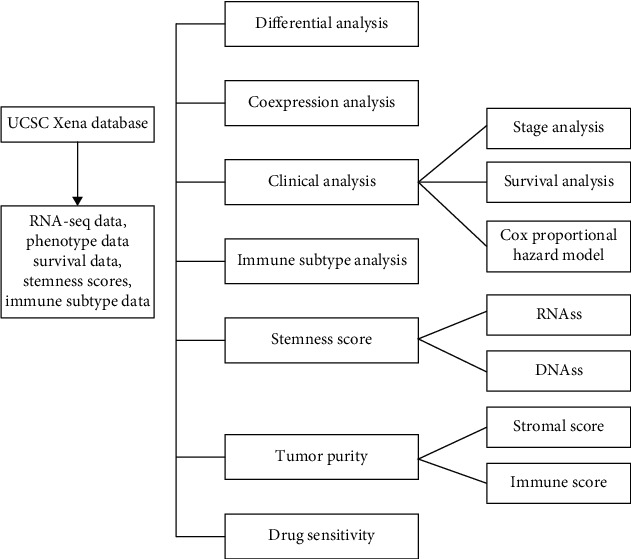
The flowchart of the present study.

**Figure 2:**
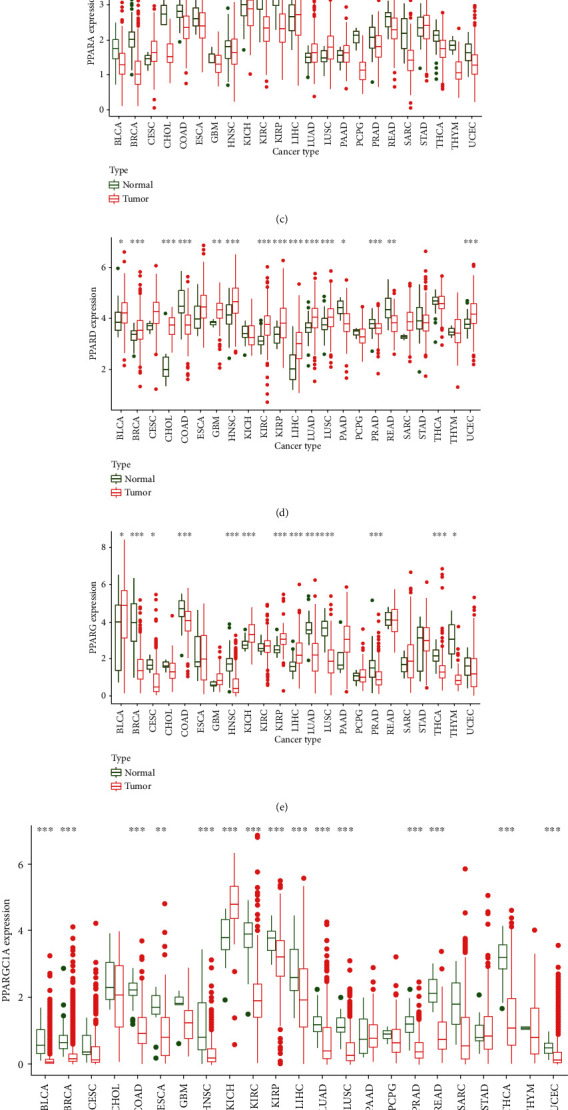
Differential expression analysis. (a) The box plot showing the transcriptional expression levels of PPARs. (b) The heatmap showing the transcriptional level of PPARs in TCGA tumor types compared to normal tissues; the gradient colors represent the log Fold Change (logFC) value. (c–g) The box plots showing differential expression of PPARs and PPARGC1A and PPARGC1B in normal and tumor tissues (^∗∗∗^*p* < 0.001; ^∗∗^*p* < 0.01; ^∗^*p* < 0.05); (h) the correlation coefficients by coexpression analysis between every two genes are presented.

The flowchart of the analysis process is summarized in Figure 1. The gene expression of PPARA, PPARD, PPARG, PPARGC1A, and PPARGC1B was displayed (Figure 2(a)). Differential expression analyses with the Wilcox test were performed on 5 PPAR family genes between tumor and paratumor samples (Figure 2(b)). Those 5 PPAR genes were either down- or upregulated in most types of tumors. PPARA, PPARG, PPARGC1A, and PPARGC1B were seen with low expression in the majority of tumors while PPARD is mainly upregulated.

Specifically, compared to normal tissues, PPARA was observed with low expression in most types of tumors except pan-lung: LUAD and LUSC. It is also obvious that PPARA was the only gene in the PPAR family that was downregulated in CHOL (*p* < 0.001, Figure 2(c)). Interestingly, however, we found significant overexpression of PPARD in CHOL (*p* < 0.001, Figure 2(d)). There was a significantly differential expression of PPARG in BRCA. More than BRCA, both two lung tumors, LUAD and LUSC, expressed low PPARG (*p* < 0.001), which is opposite to PPARA as well as PPARD and different from the other 4 PPAR family genes (Figure 2(e)). Significant overexpression of PPARGC1A was observed in KICH (*p* < 0.001), and downregulation was observed in KIRC and THCA (*p* < 0.001) (Figure 2(f)).

We also queried PPAR protein expressions from the Human Protein Atlas database (https://www.proteinatlas.org), and the PPAR proteins that combined to specific antibodies in both tumor and normal issues were displayed in Figure S1, which tend to follow the same expression patterns as the results of differential expression analysis.

Coexpression analysis revealed a correlation (correlation coefficient = 0.45) between PPARA and PPARGC1A, suggesting a potential positive interaction between those two genes (Figure 2(h)), which was further confirmed by the protein-protein interaction (PPI) network (Figure S2). The coexpression relationship could also be observed between PPARA and PPARG (correlation coefficient = 0.24, *p* < 0.001). By contrast, a different coexpression pattern was seen between PPARGC1A and PPAGC1B with a negative correlation (correlation coefficient = −0.13, *p* < 0.001).

### 3.2. Clinical Correlation Analysis

**Figure 3:**
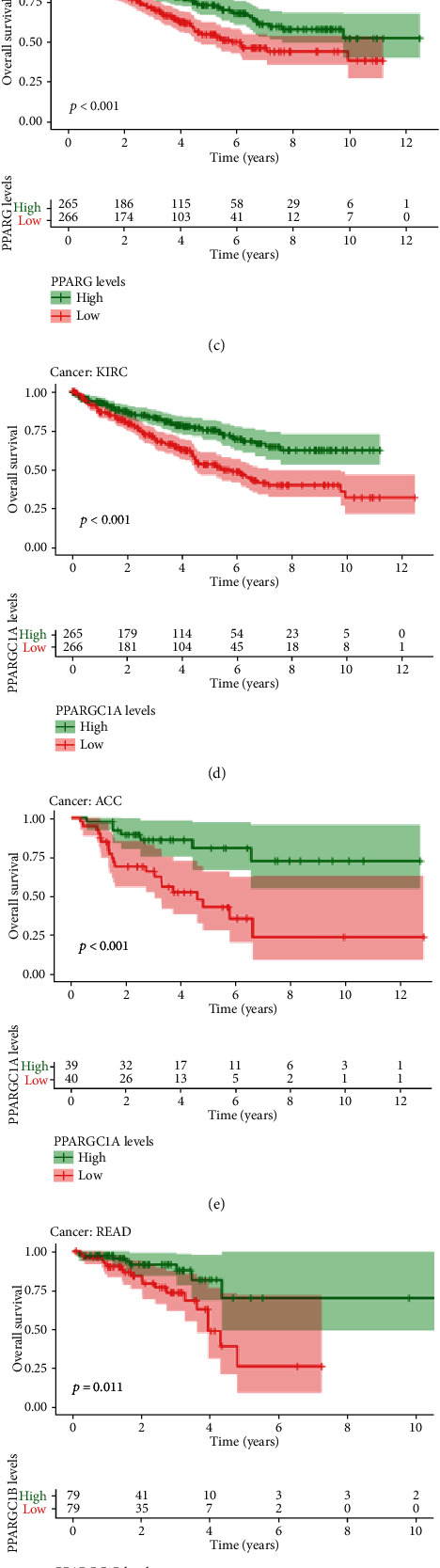
The results of survival analysis of PPARs in pan-cancer. (a–f) Kaplan-Meier plots of PPARs in pan-cancer showing the differential survival outcomes of high PPAR and low PPAR (*p* < 0.05). (g) Cox proportional hazard analyses illustrating the hazard ratios (HRs) of PPARs in 33 TCGA tumors; those PPARs whose HR > 1 in certain types of cancer were regarded as danger factors of the very type of cancer, which were unfavorable for prognostic outcomes.

We employed Kaplan-Meier analyses on PPARA, PPARD, PPARG, PPARGC1A, and PPARGC1B in 33 TCGA tumors (Figures 3(a)–3(f)). Based on the median gene expression values, patients were divided into high and low groups.

Low expression of PPARA was significantly associated with poor prognosis in patients with KIRC (*p* < 0.01, Figure 3(a)), GBM (*p* = 0.026), and LGG (*p* = 0.009).

By contrast, elevated expression of PPARD was correlated with worse clinical outcomes of patients with LGG (*p* = 0.040), LIHC (*p* = 0.018), and SARC (*p* = 0.011) while elevated PPARD led to better clinical outcomes in BLCA (*p* = 0.025) and UVM (*p* = 0.006).

The higher expression of PPARG and PPARGC1A was associated with better prognostic outcomes in KRIC (*p* < 0.001, Figures 3(c) and 3(d)). Likewise, low expression of PPARGC1B might be a less favorable sign for clinical outcomes in patients of READ (*p* = 0.011, Figure 3(f)), which is consistent with the differentially low expression in READ compared to paratumor samples.

Cox proportional hazard regression was applied to detect the prognostic roles of PPARA, PPARD, PPARG, PPARGC1A, and PPARGC1B in 33 TCGA tumors. Genes with a hazard ratio (HR) > 1 were considered as a prognostic factor. From the forest plot (Figure 3(g)), we found that PPARD and PPARG were of pan-cancer significance with HR > 1 in most cancer types.

**Figure 4:**
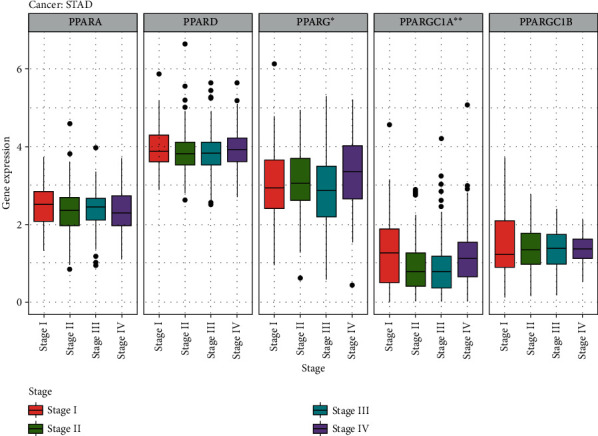
Differential gene expression level of PPARs in different tumor stages in STAD (PPARG: *p* < 0.05; PPARGC1A: *p* < 0.01).

Specifically, in STAD, we found that the expression of PPARG (*p* = 0.016) and PPARGC1A (*p* = 0.005) was correlated with TNM stages. The expression level of PPARG was comparatively lower in stage III and higher in stage IV. Compared to other TNM stages, the expression level of PPARGC1A was the highest in stage I, followed by stage IV, and was comparatively low in stage II and stage III (Figure 4). The difference in the expression level of PPAR family genes in different TNM stages might serve as predictors of tumor development in clinical applications.

### 3.3. Immune Subtype Analysis

**Figure 5:**
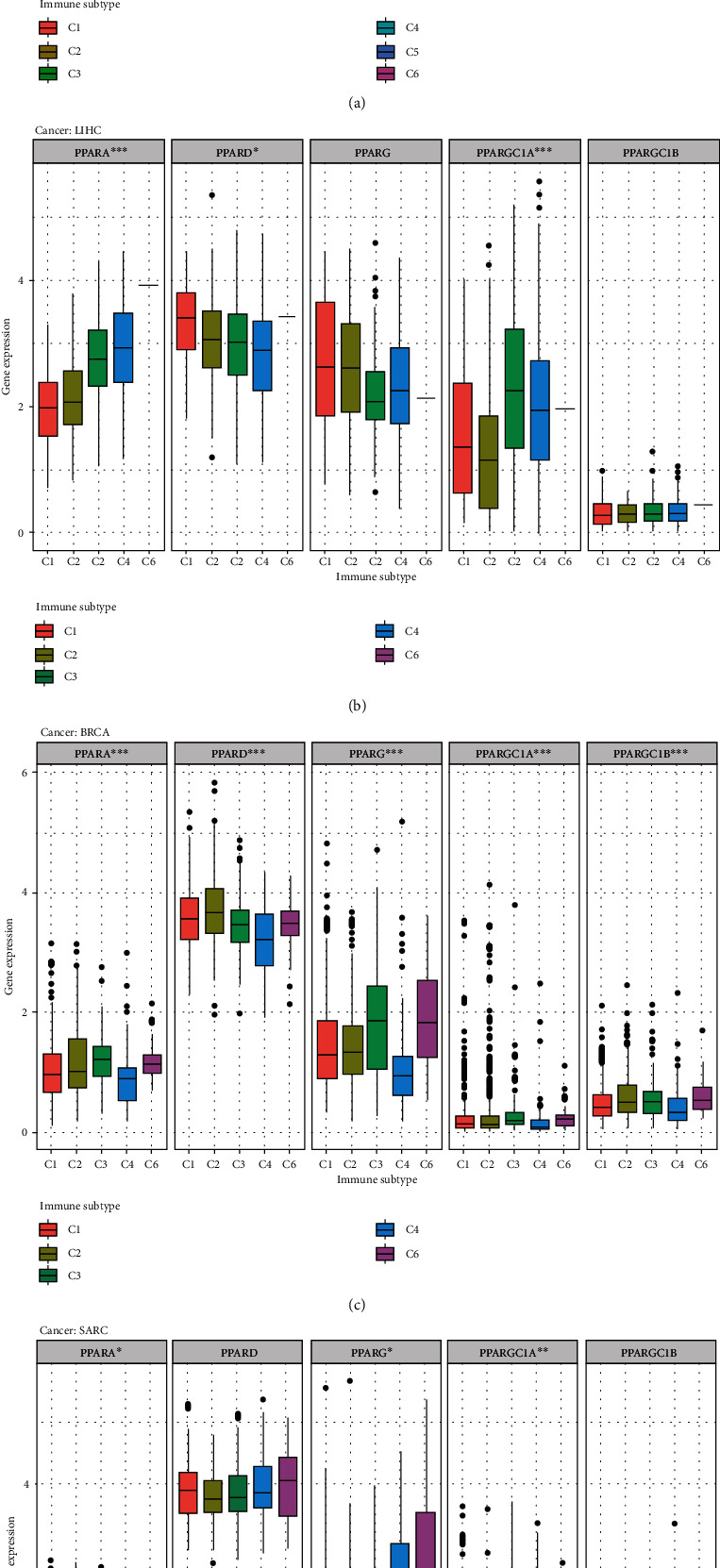
The results of correlation analysis between members of PPAR and immune subtypes. (a) The transcriptional expression of PPARs in C1-C6 immune subtypes across TCGA cancers. (b–d) Box plots showing the expression level of PPAR immune subtypes in LIHC, BRCA, and SARC, respectively (^∗∗∗^*p* < 0.001; ^∗∗^*p* < 0.01; ^∗^*p* < 0.05). LIHC: liver hepatocellular carcinoma; BRCA: breast invasive carcinoma; SARC: Sarcoma.

We applied differential expression analysis with the Kruskal test on the mRNA expression of 5 PPAR genes in the six immune subtypes across 33 TCGA tumor types (Figure 5(a)).

The expression patterns of PPARA (*p* < 0.001), PPARD (*p* < 0.001), PPARG (*p* < 0.001), PPARGC1A (*p* < 0.001), and PPARGC1B (*p* < 0.001) varied in 6 immune subtypes in pan-cancers (Figure 5(a)). Obviously, PPARD ranked the first on the overall expression level in C1-C6.

In addition, different types of tumors displayed variation within immune subtypes. For the C1-C6 immune subtypes of LIHC, there were differences in the expression of PPARA (*p* < 0.001), PPARD (*p* < 0.05), and PPARGC1A (*p* < 0.01) (Figure 5(b)). C6 had the highest expression of PPARA, followed by C4 and C3 while C4 has the lowest expression of PPARD. The expression level of PPARGC1A varied by immune subtypes, with C3, C4, and C6 comparatively high whereas C1 and C2 low.

In BRCA, significant differences were observed in the expression of PPAR family genes in the six immune subtypes (Figure 5(c)). In general, C4 has the lowest expression of PPAR genes. The expression of PPARA (*p* < 0.001), PPARG (*p* < 0.001), PPARGC1A (*p* < 0.001), and PPARGC1B (*p* < 0.001) showed similar patterns in C1-C6, with high expression in C3 and C6 while comparatively low expression in C1, C2, and C4. PPARD, however, expressed higher in C1 and C2 compared to other immune subtypes.

For SARC, C6 had the lowest expression of PPARA (*p* < 0.05) and PPARGC1A (*p* < 0.01), whereas the expression level of PPARG (*p* < 0.05) was the highest among C1, C2, C3, C4, and C6 immune subtypes (Figure 5(d)).

### 3.4. Stemness Indices and Microtumor Environment in Pan-Cancer

**Figure 6:**
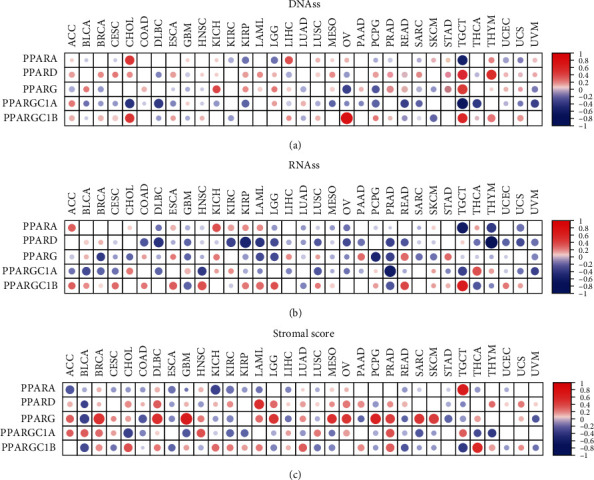
The results of correlation analysis between members of PPAR and stemness indices and microenvironment scores. (a, b) The two heatmaps showing the correlation of the expression level of PPARA, PPARD, PPARG, PPARGC1A, and PPARGC1B and stemness indices (DNAss and RNAss) in 33 TCGA cancer types. DNAss: DNA methylation-based stemness score; RNAss: RNA-based stemness score. (c) The heatmap showing the correlation between stromal scores and the mRNA expression of PPARs (red points represent a positive correlation while blue points represent a negative correlation).

Stromal scores of TCGA cancer samples were calculated by applying the ESTIMATE (Estimation of STromal and Immune cells in MAlignant Tumors using Expression data) algorithm [11]. Spearman correlation analysis was used to describe the correlation between the expression level of PPAR family genes and stromal scores in pan-cancer. As is shown in Figure 6(c), we found a positive correlation between PPARA and stromal scores in TGCT (correlation coefficient = 0.60, *p* = 0). There was likewise a relationship between PPARD and LAML with a correlation coefficient = 0.48, *p* < 0.001. The expression of PPARG was positively correlated with a number of tumor types, including BRCA, DLBC, LGG, MESO, OV, PCPG, PRAD, SARC, and SKCM, suggesting that elevated expression of PPARG was associated with lower tumor purity in many types of tumors. Significant differences were found between PPARGC1A and PPARGC1B towards their relationship with tumor purity. The higher expression of PPARGC1A was correlated with high tumor purity in CHOL, GBM, KIRC, KIRP, and THCA, while with low stromal scores of BLCA, HNSC, LUSC, and TGCT, which was the opposite to the pattern of PPARGC1B.

To analyze the correlation between PPARs and stemness features of pan-cancer, we calculated the stemness indices of TCGA tumor samples by using a one-class logistic regression (OCLR) algorithm and performed Spearman correlation analysis based on gene expression and stemness scores [12]. Two types of stemness indices were accessed, which included DNA methylation-based stemness index (DNAss) and mRNA expression-based stemness index (RNAss).

There were differences between the two stemness indices on the correlation with the PPAR expression level in TCGA tumors. For DNAss, it is obvious that there were strong correlations between TGCT and PPAR family genes, with positive correlations of PPARD (correlation coefficient = 0.56, *p* < 0.001), PPARG (correlation coefficient = 0.44, *p* < 0.001), and PPARGC1B (correlation coefficient = 0.52, *p* < 0.001) and negative correlations of PPARA (correlation coefficient = −0.59, *p* < 0.001) and PPARGC1A (correlation coefficient = −0.66, *p* < 0.001) (Figure 6(a)).

For RNAss, strong negative correlations were observed between TGCT RNAss and PPARA (correlation coefficient = −0.63, *p* < 0.001), between THYM RNAss and PPARD (correlation coefficient = −0.81, *p* < 0.001), between PCPG RNAss and PPARG (correlation coefficient = −0.53, *p* < 0.001), and between PRAD RNAss and PPARGC1A (correlation coefficient = −0.63, *p* < 0.001) (Figure 6(b)). A positive association between the expression profiles of PPARGC1B and the RNAss of TGCT was detected (correlation coefficient = 0.64, *p* < 0.001), suggesting that PPARGC1B might correlate with the stemness in TGCT.

**Figure 7:**
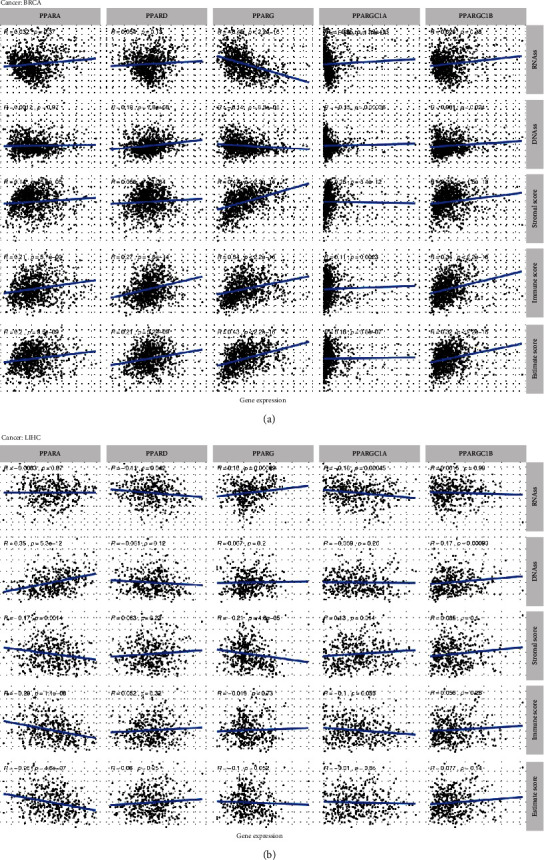
The correlation between PPARs and their coactivators and stemness scores (RNAss and DNAss), stromal scores, immune scores, and ESTIMATE scores in breast invasive carcinoma (BRCA) and liver hepatocellular carcinoma (LIHC).

In BRCA (Figure 7(a)), specifically, the expression profiles of PPARA was positively correlated with BRCA stromal scores (correlation coefficient = 0.14, *p* < 0.001), immune scores (correlation coefficient = 0.21, *p* < 0.001), and ESTIMATE score (correlation coefficient = 0.2, *p* < 0.001). The expression profiles of PPARD were positively correlated with BRCA DNAss (correlation coefficient = 0.19, *p* < 0.001), immune scores (correlation coefficient = 0.27, *p* < 0.001), and ESTIMATE score (correlation coefficient = 0.21, *p* < 0.001). Notably, we found negative correlations between PPARG expression with RNAss (correlation coefficient = −0.45, *p* < 0.001) and DNAss (correlation coefficient = −0.14, *p* < 0.001) while positive correlations with BRCA's stromal score (correlation coefficient = 0.47, *p* < 0.001), immune score (correlation coefficient = 0.34, *p* < 0.001), and ESTIMATE score (correlation coefficient = 0.43, *p* < 0.001). In addition, slight but statistically significant correlations were found between PPARGC1A and stemness indices and tumor purity. There were strong correlations, however, between PPARGC1B and stromal score (correlation coefficient = 0.23, *p* < 0.001), immune score (correlation coefficient = 0.34, *p* < 0.001), and ESTIMATE score (correlation coefficient = 0.32, *p* < 0.001).

For LIHC (Figure 7(b)), however, there were slight correlations between each PPAR family gene and stemness indices and TME except relatively strong associations between PPARA and tumor purity (stromal score: correlation coefficient = −0.17, *p* < 0.001; immune score: correlation coefficient = −0.29, *p* < 0.001; and ESTIMATE score: correlation coefficient = −0.26, *p* < 0.001).

### 3.5. Drug Sensitivity Analysis in Pan-Cancer

To analyze the potential effects of the PPAR family on drug response, we performed Pearson correlation analysis between the transcriptional expression of PPAR family genes in NCI-60 cancer cell lines and drug activity of 263 antineoplastic drugs retrieved from the CellMiner database [16].

**Figure 8:**
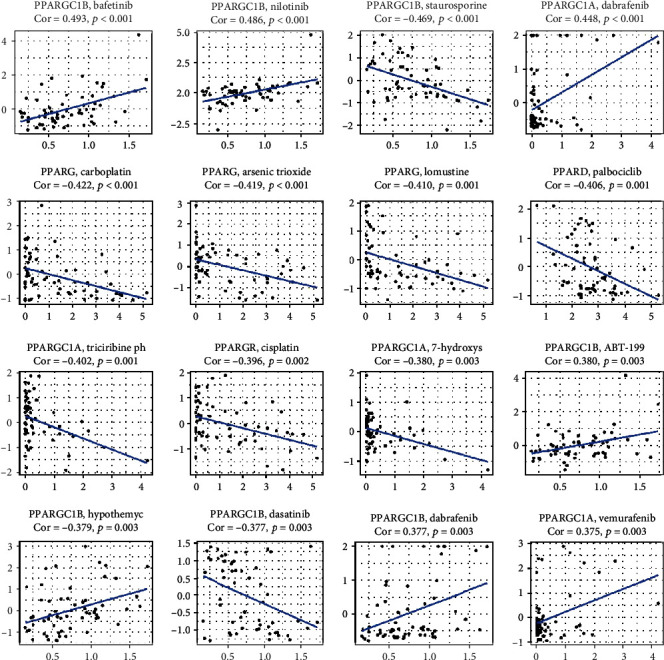
Drug response analysis. The correlation between drug sensitivity and PPARA, PPARD, PPARG, PPARGC1A, and PPARGC1B across TCGA cancers. The scatter plots are ranked by *p* value.

The scatter plots that displayed a significant correlation relationship between drug sensitivity and gene expression are presented in Figure 8 and ranked by the *p* value, selected by *p* < 0.05. Notably, PPARGC1B was positively correlated with the sensitivity of Bafetinib (correlation coefficient = 0.493, *p* < 0.001) and Nilotinib (correlation coefficient = 0.486, *p* < 0.001) and the resistance of staurosporine (correlation coefficient = −0.469, *p* < 0.001). The sensitivity of dabrafenib, a selective inhibitor of mutated forms of BRAF kinase for BRAF-mutated melanoma, thyroid cancer, and non-small-cell lung cancer, was found to be positively associated with PPARGC1A (correlation coefficient = 0.448, *p* < 0.001) and PPARGC1B (correlation coefficient = 0.377, *p* = 0.003). Highly expressed PPARG tumor cells were more resistant to carboplatin (correlation coefficient = −0.422, *p* < 0.001), cisplatin (correlation coefficient = −0.396, *p* = 0.002), arsenic trioxide (correlation coefficient = −0.419, *p* < 0.001), and lomustine (correlation coefficient = −0.410, *p* = 0.001) (Figure 8).

## 4. Discussion

In the present study, we aimed to explore the correlation of PPAR transcriptional expression with TCGA tumor features, which include TME, clinical significance, immune subtypes, stemness, and drug responses. PPAR isotypes showed distinct effects on tumor development. Using multidimensional analysis, we first performed differential expression analysis on a total of 11,057 samples (10,327 tumor samples and 730 adjacent samples) across 33 TCGA cancer types and found significant difference on the PPARs' expression level in different tumor types. We also applied survival analysis and Cox proportional hazard regression. Statistically significant survival differences were observed between high and low PPAR-expressed patients in some types of cancers, suggesting that PPARs might become potential prognostic indicators for clinical applications.

It is also worth noting that PPARG along with PPARGC1A was found to be differentially expressed in the 4 stages of stomach adenocarcinoma, with highest PPARG in stage IV, which is consistent with the findings of Nagy et al. that PPARG may contribute to STAD carcinogenesis [17]. In this study, however, PPARG was found to have low expression in the majority of TCGA cancers. Evidence backed for PPARG's antineoplastic actions in inducing cell cycle arrest, terminal differentiation, and anti-inflammatory effect [18]. Troglitazone (TGZ), a PPARG agonist, was reported to induce G2/M cell cycle arrest through activation of p38 mitogen-activated protein kinase in renal cell carcinoma [18, 19], and similar effects were also seen in bladder cancer cells [20]. Another agonist of PPARG, curcumin, was able to eliminate oxidative stress and chronic inflammation via downregulating the WNT/*β*-catenin pathway, which is observed to have aberrant activation in many cancers [21]. Sporadically, the tumor-promoting side of PPARG was observed in some cancers; it is easy to infer that the precise effects of PPARG and its agonists might depend on types of cancers and tumor environment.

Moreover, according to the C1-C6 immune subtypes previously identified by investigators [10], we classified tumor samples by representative immune signatures and examined the RNA-seq level of PPARA, PPARD, PPARG, PPARGC1A, and PPARGC1B from C1 to C6, which were all seen to have differential expressions. These immune features along with extracellular matrix, tumor vasculature, and tumor cells make up the concept of the tumor microenvironment (TME), the heterogeneity of which highly influences therapeutic response and clinical prognosis [22]. Thus, we further accessed the fractions of stromal cells and immune cells in tumor samples of 33 TCGA cancer types by calculating stromal scores, immune scores, and ESTIMATE scores. Those TME characteristics were correlated with the expression level of PPARA, PPARD, PPARG, PPARGC1A, and PPARGC1B. Unexpectedly, correlations did exist in some types of cancers. In breast invasive carcinoma, particularly, PPARG and PPARGC1B were negatively correlated with tumor purity.

Stemness has been proposed to describe the stem cell-like characteristics of the tumor: self-renewal and dedifferentiation [23]. The acquisition of stem cell-like properties has been reported to be found in many tumor progression [24]. Here, we utilized an OCLR approach to calculate the RNAss score and DNAss score of tumor samples and then correlated it with transcriptional signatures of PPARs. We found an association between PPARs and stemness within tumors, suggesting that PPARs may play a role in stemness maintenance.

This study also found that the transcriptional expression level of PPARs, PPARG1A, and PPARG1B was associated with drug responses. Notably, high expression of PPAGC1B was even more sensitive to Bafetinib and Nilotinib across cancer treatments, which is of clinical significance for selection of antitumor therapies.

The three isotypes of peroxisome proliferator-activated receptors differ in both physiological functions and roles in carcinogenesis. PPAR*α*, encoded by PPARA, mainly enriches in the liver, kidney, and heart, regulating fatty acid metabolism and mitochondrial biosynthesis [25]. In addition to its endogenous ligands (fatty acids), PPAR*α* responds to the PPAR*α* agonists (synthetic fibrates), such as fenofibrate and gemfibrozil, which have been working well in the treatment for hypolipidemic diseases [26]. Moreover, PPAR*α* agonists have been reported to show antitumor effects in colon carcinogenesis. However, it is still controversial whether the roles of PPAR*α* is cancer-repressing or cancer-promoting [25]. Some studies suggested that long-term activation of PPAR*α* induced hepatocellular carcinoma in mice and was essential for the development of hepatic steatosis [27]. The roles of PPAR*α* in carcinogenesis require further elucidation. PPARG, encoding PPAR*γ*, functions as a key regulator of glucose homeostasis and adipocyte differentiation [28]. Downregulation of PPAR*γ* is associated with decreased terminal differentiation and cell cycle arrest, which induces cell proliferation and leads to tumorigenesis [7, 29]. The potential mechanism was proposed by Drori et al. that the PPAR*γ*-induced differentiation may be mediated by a putative PPAR*γ* coactivator, HIC5, suggesting the importance of coactivators in PPAR*γ* signaling [30]. Peroxisome proliferator-activated receptor coactivators 1 alpha and beta (PPAGC1A and PPARGC1B, respectively) cooperate with PPARP*γ*, allowing the subsequent interaction between PPAR*γ* and other transcription factors [31, 32]. Pharmacological activators of PPAR*δ* also show controversial effects on the hallmarks of caner, which may depend on the type of PPAR*δ* ligands and target tissues [33, 34].

Although this study is the first one to multidimensionally analyze peroxisome proliferator-activated receptors (PPARs) in pan-cancer, it still possessed some limitations that warrant consideration. Firstly, all the samples involved in this study were from America, and thus, we were not quite sure about the applicability of the prediction model in Europe and Asia. Second, the results of this study have not been verified by other independent databases, and thus, our future work is validating it by our own data and other public database. Third, the potential mechanism in this study is based on bioinformatics analysis and has not been verified by molecular and animal experiments. The analysis of this study focuses on the correlation between the PPAR family and multiple omics data. However, the biostatistical correlation could not elucidate the direct interaction and direct regulation mechanism, which should be the main limitation of this study. Thus, we plan to verify these potential mechanisms via molecular experiments. Further investigations are required to figure out the potentials of PPARs and their coactivators as drug targets for cancer, which makes our study even more important in the contribution to the expression signature analysis of PPARA, PPARD, PPARG, PPARGC1A, and PPARGC1B.

## 5. Conclusion

We performed multidimensional analyses on PPARA, PPARG, PPARD, PPARGC1A, and PPARGC1B, including differential expression analysis in pan-cancer, immune subtype analysis, clinical analysis, tumor purity analysis, stemness correlation analysis, and drug responses. PPARs and their coactivators expressed differently in different types of cancers, in different immune subtypes. This analysis reveals various expression patterns of the PPAR family at a level of pan-cancer and provides new clues for the therapeutic strategies of cancer.

## Data Availability

The datasets generated and/or analyzed during the current study are available in Supplementary Materials and TCGA program (https://portal.gdc.cancer.gov).

## Supplementary Materials

Supplementary Materials Figure S1: PPAR protein expression in cancer (breast cancer, colon adenocarcinoma, and prostate cancer) and normal tissues from the Human Protein Atlas database. Figure S2: the protein-protein interaction (PPI) network constructed among PPARA, PPARD, PPARG, PPARGC1A, PPARGC1B, and their top related genes.
