# Supplementary material for: The Role of Peroxisome Proliferator-Activated Receptors (PPARs) in Pan-Cancer
Source: PPAR Res. 2020 Sep 22;2020:6527564. doi: 10.1155/2020/6527564 (PMC7528029; doi:10.1155/2020/6527564)
Supplement: Supplementary Materials — Figure S1: PPAR protein expression in cancer (breast cancer, colon adenocarcinoma, and prostate cancer) and normal tissues from the Human Protein Atlas database. Figure S2: the protein-protein interaction (PPI) network constructed among PPARA, PPARD, PPARG, PPARGC1A, PPARGC1B, and their top related genes. [file 6527564.f1.pdf]

Supplementary Materials

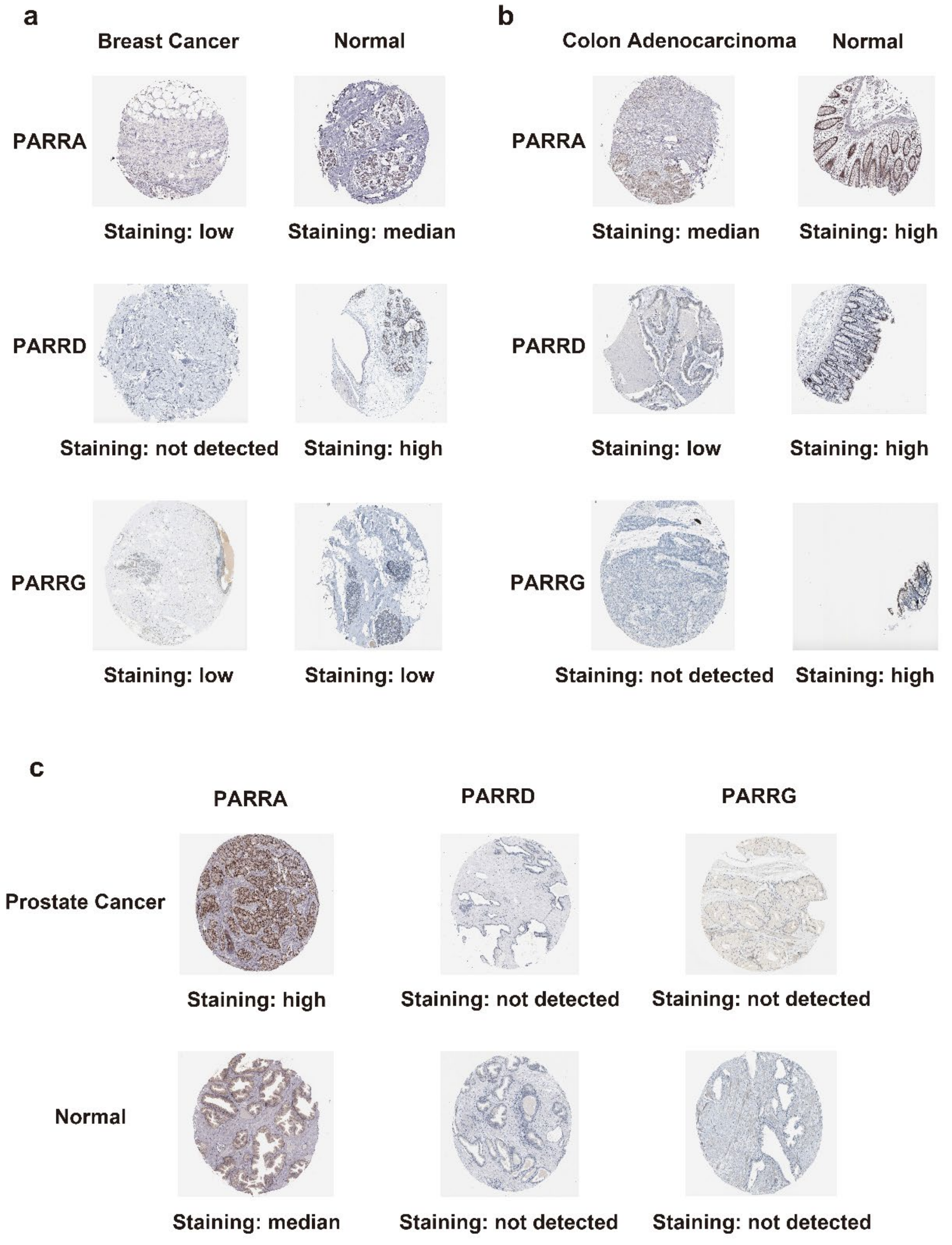

Figure S1 PPAR protein expression in cancer (breast cancer, colon adenocarcinoma and prostate cancer) and normal tissues from the human protein atlas database

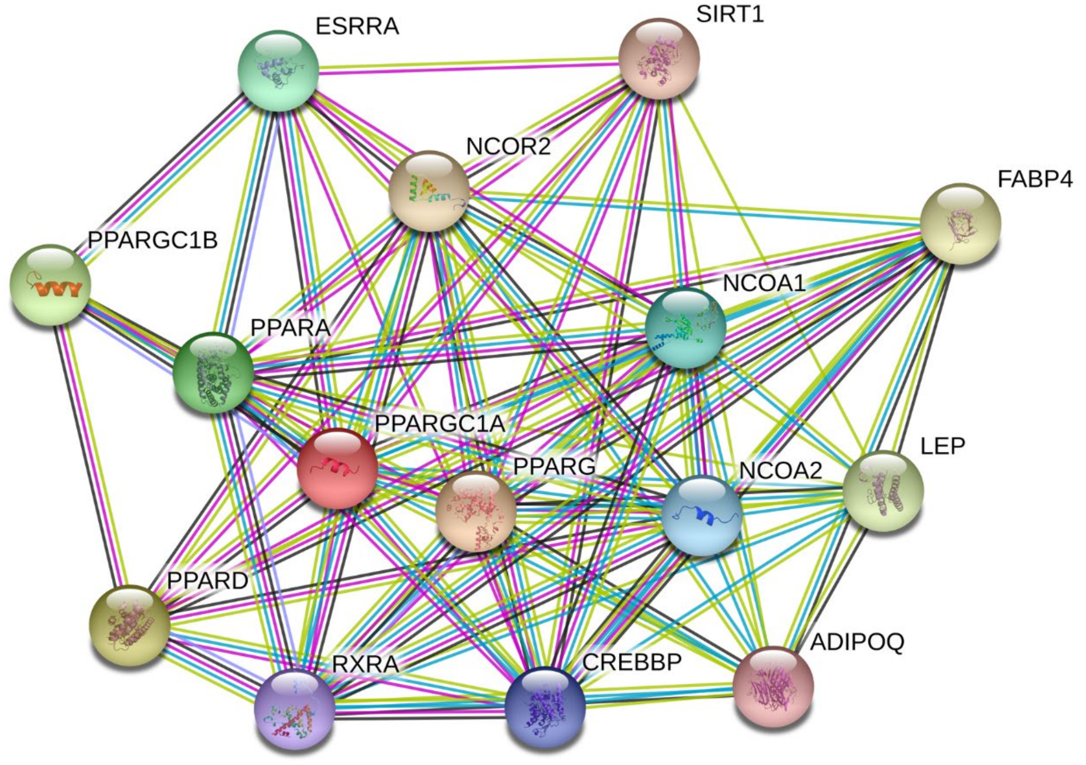

Figure S2 The protein-protein interaction (PPI) network constructed among PPARA, PPARD, PPARG, PPARGC1A, PPARGC1B and their top related genes.
